# Sexual experiences among multicultural adolescents in Korea: evidence from the Korean Youth’s Risk Behavior Survey

**DOI:** 10.3389/fsoc.2024.1353304

**Published:** 2024-03-12

**Authors:** Keuntae Kim, Se Hee Jo

**Affiliations:** ^1^Department of Public Sociology, Korea University Sejong Campus, Sejong, Republic of Korea; ^2^Social Science Research Institute, Busan National University, Busan, Republic of Korea

**Keywords:** adolescents, sexual intercourse, multicultural background, KYRBS, Korea

## Abstract

Several studies have examined adolescent sexual behaviors by family immigration status, but most of these failed to account for heterogeneity within youths’ multicultural backgrounds. To fill this gap in the literature, this paper draws data from the 2011 to 2022 rounds of the Korean Youth’s Risk Behavior Survey (*N* = 769,160) and compares the likelihood of sexual intercourse across four groups of adolescents. Results from logistic regression indicate that the odds of having sexual contact increased 2.8 times for youths with a non-Korean father and Korean mother, compared with those from families with two Korean parents. When both father and mother are foreign-born, the odds of being sexually active increased 4.7 times. In both cases, the discrepancies might be primarily associated with the foreign fathers’ lack of socioeconomic resources. Therefore, the father’s role deserves more examination, and sex education in schools should be tailored to reflect multicultural adolescents’ needs.

## 1 Introduction

With an influx of foreign workers and marriage migrants since mid-1990s, Korea is rapidly transforming into a multicultural society. This transformation has created an increase in multicultural families over the past decade, resulting in a growing proportion of multicultural youths at all school levels (Ministry of Education, 2022). Unfortunately, evidence from recent research suggests that a significant proportion of multicultural adolescents experience racial prejudice, difficulties in communicating with friends and family members, and challenges in adapting to Korean culture (Seol, 2014). Also, the average socioeconomic status of multicultural families is substantially lower than that of the native population, and this gap is larger than the gaps found in other East Asian countries, such as Japan, Taiwan, or Singapore (Kim, 2010, 2021; Chung et al., 2016).

Against this backdrop, sexual desire typically grows stronger during adolescence, as does the importance of relationships with the opposite sex, but many adolescents do not have adequate sexual consciousness or attitudes: evidence from past literature suggests that, on average, early sexual experience during adolescence can be directly and indirectly associated with numerous negative outcomes, such as substance use (Brooks-Gunn and Furstenberg, 1989; Lee et al., 2018), lower educational aspirations (National Research Council, 1987; Kim, 2020), and risky health behaviors (Mota et al., 2010). Moreover, earlier sexual debut would increase the likelihood of unintended pregnancy and childbearing, which might hamper socioeconomic achievement over the life course (Kim, 2014). In addition, a large body of research found that adolescents with lower socioeconomic status are positively associated with earlier sexual initiation (Crockett et al., 1996; Miller et al., 1999). Taken together, the increased cultural diversity in Korea and the fraught patterns of adolescent sexual outcomes strongly suggest that a growing number of multicultural adolescents in Korea need policy interventions that are related to sexual behaviors and development.

However, our understanding of sexual behaviors and its determinants among multicultural Korean adolescents is quite limited. A few studies (Lee et al., 2015; Nam, 2015; Son and Choi, 2020) addressed sexual behaviors among multicultural adolescents by comparing them to native students, but one issue with this research is that they tend to view multicultural adolescents as a homogeneous group – probably because their main objective is to highlight the magnitude of sexual activities relative to Korean natives. Obscuring the variations within multicultural adolescents may impede our understanding of the mechanisms of social disadvantages faced by immigrants and their families in Korea. Another issue with past research in this content area is that it relies on cross-sectional analyses, which precludes examination and understanding of trends in the risk factors for multicultural adolescents’ sexual contacts.

To bridge this gap in the literature, this study explores the determinants of sexual intercourse among adolescents from varied multicultural backgrounds, and examines trends in the likelihood of sexual contact by multicultural status. These types of analyses would help develop more effective policies for facilitating assimilation of immigrants and their children in Korea.

## 2 Background

### 2.1 Multicultural adolescents in Korea

It is well documented that the share of foreign-born people in Korea has been increasing rapidly since the mid-2000s. According to the Ministry of the Interior and Safety (2022), the first statistics on the number of registered foreigners was made available in 2006 and the share at that time was only 1.1%. As of 2021, that share nearly quadrupled to 4.1%. One of the principal drivers for this expansion is an influx of marriage migrants, most of whom are females from Southeast Asian countries marrying Korean men (Kim, 2017).

**Figure 1:**
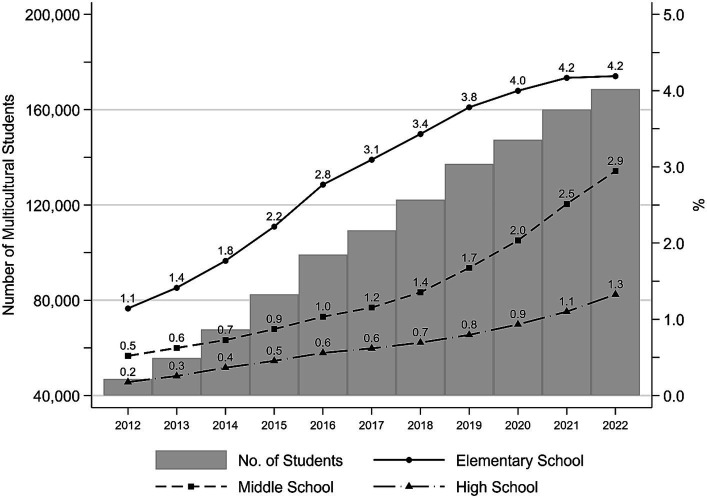
Trends in the number of multicultural students and proportions of multicultural students in elementary, middle, and high schools in Korea. Multicultural students include both those from immigrant families and those who were born in Korea but one of their parents is not a Korean. *Data*: 2022 Basic Statistics on Education (Ministry of Education, 2022).

Because the average marriage migrant tends to give birth shortly after the marriage (Kim, 2021), children born to internationally married couples in the early 2000s are now teenagers, and their proportion increase parallel those of the overall foreigners. As shown in Figure 1, both the absolute number and the proportions of multicultural adolescents in all school levels (elementary, middle, and high school) have consistently risen since the early 2010s. The total number of multicultural students almost quadrupled from nearly 47,000 in 2012 to nearly 169,000 in 2022 (Ministry of Education, 2022). Over this same time period, the percent of multicultural children in elementary schools increased from 1.1 to 4.2%, the fraction of multicultural middle school students changed from 0.5 to 2.9%, and multicultural high schoolers rose from 0.2 to 1.3%.

While the percentages of multicultural students might decrease in a particular year as the school level rises, this could be attributed to the fact that multicultural children are disproportionately younger than their native peers. Hence, as children in elementary schools enter upper-level schools, the share of multicultural adolescents is expected to increase for the foreseeable future.

### 2.2 Antecedents of sexual intercourse among adolescents

It appears that there is no overarching theoretical framework that substantially explains either adolescents’ sexual initiation or its intersections with the multicultural backgrounds (Crockett et al., 1996). However, there is little dispute that adolescence is a physical and psychological transitional period from childhood to adulthood, and that experiencing rapid change and growth often leads to confusion about self-identity (Brooks-Gunn and Furstenberg, 1989). Also, adolescents may encounter various social barriers when they try to use their newly-developed physical and psychological capacities – largely because most of them have not achieved full-fledged independence like adults. Nevertheless, some adolescents break through these barriers by engaging in behaviors such as sexual intercourse, smoking, or drinking alcohol, which are often referring to as deviant behaviors (Benda and DiBlasio, 1994).

A large body of research has repeatedly and consistently found that the family environment has a strong influence on an adolescent’s sexual activity. For example, it appears that parent’s educational attainment delays the onset of a youth’s sexual activities (Forste and Heaton, 1988). This can be partially attributable to the notion that more-educated parents tend to put greater values on teens’ academic performance and educational aspiration, which might be positively associated with an adolescent’s intellectual achievement and, in turn, impede sexual initiation. Another partial explanation is that parents’ educational level is closely associated with socioeconomic resources, which can be used to control an adolescent’s behaviors when conflicts between parents arise (Goldscheider and Goldscheider, 1993).

Another type of family effect on adolescents’ sexual activity is parental controls. According to the parental control hypothesis, parents not only teach children the norms and values of society (i.e., socialization), but also can suppress teen’s deviant behaviors through setting rules, supervision, and surveillance (Miller and Fox, 1987). Hence, this hypothesis suggests that two parents would be better able to monitor their adolescents’ sexual activities than single parents.

Nevertheless, even the most attentive parents may not be able to monitor all conduct, and the parent–child bond theory suggests that adolescents are intrinsically deviant, and are likely to have deviant impulse and tendencies without adequate constraints (Miller and Fox, 1987). Parent–child bond theory further underscores that deviant behaviors are engendered when bonding to social norms and groups, mainly family and parental expectations, are breached or weakened (Benda and DiBlasio, 1994): adolescents who are strongly attached to their parents are more likely to internalize parents’ values on proper behaviors and, and as a result, are less likely to engage in early sexual intercourse.

Though the empirical evidence is mixed, a large body of research suggests that family disruptions, such as parental divorce and remarriage, leads to an increased likelihood of the onset of adolescents’ sexual intercourse (Wu and Thomson, 2001). The presence of a stepparent may affect youth’s sexual activity through weakened parental control and socialization processes, while the divorce and remarriage could make an adolescent distrust parents’ authority or commitment and lead to premarital sexual behaviors.

Research suggests that the normative environment also matters for a youth’s sexual initiation (DeLamater, 1981). In general, adolescents residing in urban areas have more permissive attitudes toward sexual experiences and teens living in metropolitan areas are therefore more likely to be sexually active than their peers in small to medium cities or rural areas (Forste and Heaton, 1988). For instance, Flick (1986) reported that about 50% of adolescents aged 15 to 19 living in metropolitan areas had sexual intercourse while 40% of similar aged youths in other areas had the same experience. Moreover, past studies on the differentials in sexual behavior across school types found that teens in Korean coed schools are considerably more likely to be sexually active than their peers in single-sex schools (Hong and Kang, 2017). This might be associated with students in coeducational schools having relatively more opportunities to meet opposite-sex peers and interact with them than their counterparts in same-sex schools.

Past research suggests that racial minorities and immigrant youths tend to initiate deviant behaviors, including premarital sexual intercourse, at significantly early ages than their native peers (Hogan and Kitagawa, 1985; Blake et al., 2001; Wu and Thomson, 2001). It appears that the earlier onset of sexual behaviors among racial minorities and immigrant youths are associated with the family’s lower socioeconomic status, lower academic achievements, and the cultural norms prevalent in their residence area. In Korea, household income for multicultural families is significantly lower, and the proportion under the poverty line is much higher than native population (Kim, 2010). Moreover, parents’ educational level is significantly lower among multicultural families compared with native families (Nam, 2015). Also, one recent study found that international marriages are substantially more likely to be dissolved compared to those between Korean natives (Choi et al., 2019), which imply that multicultural youths are much more likely to experience parental divorce or remarriage than their native counterparts. Thus, it is reasonable to expect that the combination of the socioeconomic factors noted above would result in an earlier onset of sexual activities among multicultural adolescents than their native peers in Korea.

## 3 Method

### 3.1 Data

To examine sexual behaviors among a nationally-representative sample of adolescents with multicultural backgrounds, this study draws data from the Korean Youth’s Risk Behavior Survey (KYRBS), which has been conducted by the Korea Centers for Disease Control and Prevention (KCDC) every year since 2005. The KYRBS is a self-administered online survey of first grade middle school students (7th grade) to third grade high school students (12th grade) to identify the status and trend of health behaviors such as smoking, drinking, eating, and physical activity among Korean adolescents (Korea Centers for Disease Control and Prevention, 2023).

In 2005, the KCDC surveyed 58,224 students in 799 middle and high schools, identified through a two-stage stratified cluster sampling. Since then, about 60,000 to 70,000 students have participated in the KYRBS every year, including 51,850 students in the most recent year of 2022.

Though the dependent measure in this study (i.e., ever had sex) is available in all waves of the KYRBS, questions on the nationalities of parents has only been asked since 2011. Hence, in this study, only the 7th (2011) through 18th (2022) waves of the KYRBS are used in the analysis; after casewise deletion, 769,160 adolescents (male = 393,305, female = 375,855) remained in the analytic sample.

### 3.2 Measures

The dependent variable in the analysis is whether the respondent ever had sexual intercourse, with either the opposite or same sex. To estimate logistic regressions, the affirmative answer to the question was coded as 1 and otherwise coded as 0.^1^

An adolescent’s multicultural background, which is the main independent variable in this study, was classified into four categories by combining their parents’ county of birth: 1 = Korean father and Korean mother (KF + KM); 2 = foreign father and Korean mother (FF + KM); 3 = Korean father and foreign mother (KF + FM); 4 = foreign father and foreign mother (FF + FM).^2^ It should be noted that, from the 2019 wave onward, parents’ country of birth was administered only to students who agreed to answer to those questions. Starting in 2019, the share of youth who refused to answer those questions were 33.3, 21.8, 24.2, and 20.5%, respectively^3^; approximately 3% of the sample responded that they do not know the country of birth for either father or mother.

Parent’s educational attainment was measured with three categories: middle school and lower, high school graduate, and college and over. Adolescents who responded that they do not know either paternal or maternal educational level were excluded from analysis. Also, an adolescent’s intact family status was determined by combining information on the household members: the KYRBS asked about family members (father, mother, stepfather, stepmother, grandparents, brother and sister, and other) and whether each lives in the same housing with the respondent. When the respondent replied that either stepfather or stepmother lives with the youth, intact family was coded as 0, otherwise coded as 1.

The KYRBS asked the respondents to evaluate the overall economic situation of the family and place it in one of five categories: high, middle high, middle, middle low, and low. These five categories were collapsed to three (1 = low, 2 = middle, and 3 = high) by combining low and middle low, as well as combining high and middle high. Thus, higher scores on the subjective assessment of family’s economic situation corresponds to better living conditions.

The KYRBS measured the subjective evaluation of youth’s overall academic performances in the past 12 months with the same manner as the family’s economic situation. This is also collapsed to three categories (low, middle, and high) with the higher score indicating better educational accomplishment.

A respondent’s area of residence was determined by the address of the school that the youth is currently attending and placed in one of three categories: rural area, small to medium city, and metropolitan area.

Survey years indicate the calendar year that the survey was manually administered. Usually, the KYRBS was fielded between June and October each year (Korea Centers for Disease Control and Prevention, 2023).

### 3.3 Analysis

The present study employed a logistic regression with maximum likelihood estimation because the dependent variable is dichotomous (Long and Freese, 2006). Two models were estimated hierarchically to explore potential mediating effects: Model 1 included only demographic factors, such as gender, area of residence, and school grade (i.e., age), in addition to multicultural background; Model 2 added parent’s educational attainment, intact family, and subjective assessments on economic situation and academic performance.

All models controlled for survey year to capture any period effect, which might reflect, for instance, idiosyncratic increase or decrease in adolescents’ sexual activities during the COVID19 pandemic.

## 4 Results

Sample characteristics by sexual experience status are presented in Table 1. Though the vast majority of adolescents were native Koreans regardless of sexual experience status, significant differences in sexual experience among multicultural adolescents were found. For instance, while the proportion of the students with foreign fathers and Korean mothers among those who did not experience sexual intercourse was only 0.09%, those who did have sexual experience was 0.27%. Similarly, adolescents with foreign fathers and foreign mothers were far more likely to be sexually experienced (0.92%) than inexperienced (0.14%). However, the proportion of youths with Korean fathers and foreign mothers were not markedly different across sexual experience categories.

**Table 1:** Sample characteristics by sexual debut for the variables used in analysis: the Korea Youth Risk Behavior Survey (KYRBS) 2011–2021.

	Ever had sex	
	No	Yes
*Family type*		
Korean father + Korean mother	98.85	97.83
Foreign father + Korean mother	0.09	0.27
Korean father + Foreign mother	0.92	0.98
Foreign father + Foreign mother	0.14	0.92
*Gender*		
Male	47.12	68.06
Female	52.88	31.94
*Grade*		
7th	14.30	5.19
8th	15.23	6.78
9th	15.68	10.08
10th	18.50	17.60
11th	18.41	25.34
12th	17.87	35.00
*School type*		
Co-Ed	36.52	37.38
Boys’ school	30.06	45.07
Girls’ school	33.41	17.54
*Area of residence*		
Rural area	6.84	7.00
Small to medium city	44.76	43.14
Metropolitan	48.40	49.86
*Father’s education*		
Less than middle school	2.48	4.11
High school	32.18	35.77
More than college	65.34	60.12
*Mother’s education*		
Less than middle school	2.25	3.73
High school	39.02	42.18
More than college	58.73	54.09
*Intact family*		
No	1.56	4.00
Yes	98.44	96.00
*Subjective assessment of economic situation*		
Low	12.84	18.11
Middle	46.68	38.60
High	40.48	43.29
*Subjective assessment of academic performance*		
Low	28.77	40.88
Middle	29.31	25.37
High	41.92	33.75
*Survey year*		
2011	6.80	6.65
2012	6.83	5.19
2013	6.63	6.37
2014	6.55	6.00
2015	6.31	6.18
2016	6.28	5.17
2017	5.61	6.03
2018	14.72	16.87
2019	9.30	10.23
2020	10.48	9.23
2021	10.29	10.18
2022	10.19	11.90
*N*	278,452	13,207

Percentages are presented. Sample means are unweighted. Due to rounding, some proportions do not add up to 100%.

Consistent with past literature (Kim, 2020), significant gender differences in sexual experience were found. Among those who have not experienced sexual intercourse, the proportion of boys were 47.1% while the girls were 52.9%. Among those who had sexual experience were 68.1% of boys and 31.9% of girls. In other words, boys are much more likely to have sexual experiences compared to their female peers.

Consistent with past studies on adolescents’ sexual behaviors (Small and Luster, 1994; Park, 2015; Ryu, 2023), the share of respondents who had sexual experiences rose as an adolescent’s age increased. Moreover, the proportion of adolescents who have sexual experiences was significantly higher at boys-only schools, relative to co-ed or girls-only schools. These disparities across different school types might be associated with different sexual norms for males and females in Korean society: boys are more willing to show off their sexual experiences whereas girls are more reluctant to report their sexual behaviors even when answering a self-administered questionnaire.

The results of logistic regressions predicting sexual experience are presented in Table 2. Results from Model 1, which controlled for gender, age, school type, area of residence, and survey year, indicated that multicultural adolescents with foreign father and Korean mother are 3.5 times (*p* < 0.01) more likely to have sexual experiences than their peers with Korean father and Korean mother. Furthermore, the odds of having sexual experience for multicultural adolescents with Korean father and foreign mother increased 23.2% (*p* < 0.05). In the case of two foreign parents, the odds of having sexual intercourse increased by a factor of 8.0 (*p* < 0.01). These results suggest that, while the likelihood of having sexual experiences for all forms of multicultural adolescents are significantly higher than their Korean native peers, the magnitude was particularly elevated when adolescent’s father was not Korean.

**Table 2:** Coefficients from the logistic regression of sexual debut among Korean adolescents: the Korea Youth Risk Behavior Survey (KYRBS) 2011–2021.

	Model 1		Model 2	
	OR	SE	OR	SE
*Family type* (ref = Korean father + Korean mother)				
Foreign father + Korean mother	3.479***	(0.644)	2.761***	(0.531)
Korean father + Foreign mother	1.232**	(0.113)	1.060	(0.099)
Foreign father + Foreign mother	7.980***	(0.883)	4.695***	(0.562)
*Gender* (ref = male)				
Female	0.543***	(0.016)	0.561***	(0.017)
*Grade* (ref = 7th)				
8th	1.236***	(0.063)	1.196***	(0.062)
9th	1.805***	(0.086)	1.749***	(0.084)
10th	2.850***	(0.126)	2.733***	(0.122)
11th	4.186***	(0.179)	4.004***	(0.174)
12th	6.017***	(0.252)	5.794***	(0.246)
*School type* (ref = Co-Ed)				
Boys’ school	1.081***	(0.031)	1.102***	(0.032)
Girls’ school	0.677***	(0.024)	0.664***	(0.024)
*Area of residence* (ref = rural area)				
Small to medium city	0.864***	(0.032)	0.881***	(0.033)
Metropolitan	0.900***	(0.033)	0.931*	(0.035)
*Father’s education* (ref = less than middle school)				
High school	–	–	0.849***	(0.045)
More than college	–	–	0.757***	(0.042)
*Mother’s education* (ref = less than middle school)				
High School	–	–	0.839***	(0.047)
More than college	–	–	0.801***	(0.046)
*Intact family* (ref = No)				
Yes	–	–	0.400***	(0.020)
*Subjective assessment of economic situation* (ref = Low)				
Middle	–	–	0.704***	(0.019)
High	–	–	1.063**	(0.029)
*Subjective assessment of academic performance* (ref = Low)				
Middle	–	––	0.636***	(0.015)
High	–	–	0.609***	(0.014)
*Survey year* (ref = 2011)				
2012	0.761***	(0.040)	0.762***	(0.040)
2013	0.956	(0.048)	0.983	(0.049)
2014	0.929	(0.047)	0.981	(0.050)
2015	0.977	(0.049)	1.029	(0.052)
2016	0.821***	(0.043)	0.870***	(0.046)
2017	1.038	(0.053)	1.097*	(0.056)
2018	1.208***	(0.053)	1.309***	(0.059)
2019	1.285***	(0.061)	1.400***	(0.068)
2020	1.028	(0.050)	1.121**	(0.055)
2021	1.210***	(0.058)	1.336***	(0.065)
2022	1.412***	(0.066)	1.566***	(0.075)
Constant	0.023***	(0.002)	0.123***	(0.012)
*N*	291,659		291,659	
Log-likelihood	−50,205		−49,473	

***p < 0.01. **p < 0.05. *p < 0.1. Standard errors in parentheses.

As expected, girls are significantly less likely to experience sexual intercourse than their male counterparts. Other things being equal, girls are 45.7% less likely to have sexual experiences than boys. In line with past literature (Brooks-Gunn and Furstenberg, 1989), as adolescent’s age increases, the odds of having a sexual intercourse rose monotonically. For instance, 12th grade (seniors in high school) were about six times more likely to have a such experience compared to 7th grade students.

Reflecting the gender differences in coital behaviors, significant variations in sexual experiences across different school types were found. Adolescents in boys-only schools are 8.1% more likely to have sexual experiences compared with their peers in co-ed schools, whereas children in girls-only schools are 32.3% less likely to have coital experiences than those in co-eds. However, this result is not accordance with Hong and Kang (2017), which claimed that students in Korean coed schools are more likely to have sexual intercourse than those in single-sex schools. This dissonance might be related to the fact that Hong and Kang (2017) used a binary variable indicating whether the youths practiced safe sex rather than just having sexual intercourse.

With respect to the periods effects, the results suggest that, during the early 2010s, the likelihood of having sexual experiences among Korean youths remained more or less stable. However, beginning in the late 2010s (i.e., after 2015), it appears that the odds of coital experiences among adolescents increases, particularly during the COVID19 pandemic. In 2022, the odds of being sexually experienced increased by 41.2% over 2011.

Model 2 added parent’s educational attainment, intact family status, and subjective assessments on family’s economic situation and academic performances. The results indicate that, even after accounting for additional factors, coefficients for FF + KM and FF + FM did not lose statistical significance, though the coefficient for KF + FM became non-significant. These results suggest that, in the case of youths with KF + FM, their significantly higher odds of being sexually experienced relative to KF + KM is largely mediated by parents’ socioeconomic resources and academic performances. Simultaneously, the significantly elevated odds of having sexual experiences among multicultural adolescents either with FF + KM or FF + FM compared with children from KF + KM families cannot be fully mediated by household’s socioeconomic environments.

The size of the coefficients for all variables except multicultural statuses in Model 1 were reduced in Model 2, but none of them lost statistical significance. As suggested in the previous research, as their father’s educational attainment increases, the odds of an adolescent having coital experience declined. Compared with adolescents whose father completed less than a middle school education, the odds of having sexual experience for those whose father had a high school diploma was reduced by 15.1%. Among adolescents whose father had completed at least a college education, the same odds further declined by 24.3%. Similar patterns were observed for mother’s educational level. Relative to having a middle school or lower education, high school and college education reduced the odds of adolescent’s being sexually experienced by 26.1 and 29.9%, respectively.

Consistent with past studies (e.g., Brooks-Gunn and Furstenberg, 1989), being raised in an intact family reduced the odds of having sexual experiences by 60.0%. In other words, the presence of a stepfather or stepmother significantly raises adolescent’s likelihood of being sexually experienced. These might be attributable to that, on average, parental control and monitoring for adolescent’s behavior in stepfamilies is weaker than in biological parents. Also consistent with what the National Research Council (1987) found, youths who are not doing well in school are much more likely to have sexual intercourse than their counterparts with better academic performances. For example, adolescents who considered their academic performances to be in the middle are 36.4% less likely to have sexual experience than those who thought their academic ranking is low. Moreover, youths that thought their academic performance is high were 39.1% less likely to have coital experiences compared with those in low category.

However, an adolescent’s subjective assessment of their family’s economic status was associated with the likelihood of sexual experience, but in a somewhat unexpected direction. That is, while youths who perceive that their family’s economic situation is about average were 29.6% less likely to have sexual intercourse compared with those who evaluate it as low, adolescents who thought their family’s economic status is high were 6.3% more likely to have the experience than those in low status. Given the strong negative correlation between family socioeconomic status and the probability of adolescent sexual intercourse in prior research (National Research Council, 1987), this result might have two explanations. First, the adolescents in this sample may not have sufficient capacity to evaluate the relative standing of their family’s economic state objectively. Second, the genuine association between a family’s economic status and its adolescents’ sexual initiation might show a U-shape rather than a linear shape. In other words, the likelihood of sexual contacts among youths might be low in the middle, but high at the two ends of the socioeconomic spectrum.

With respect to period effects estimated by the coefficients for survey years, this study’s results align with Kim (2022), who found that the rate of sexual intercourse among Korean adolescents dropped significantly in 2020, when the COVID19 pandemic just started and very strict social distancing measured were implemented, but recovered rapidly in subsequent years when those measures were relaxed. Results in the current study also suggested that the odds of having sexual intercourse were declining gradually in the early 2010s, but rose starting in the mid-2010s until the outbreak of the COVID19 in 2020. The odds of sexual intercourse, however, increased quickly since 2021, and it marked the highest level in 2022.

**Figure 2:**
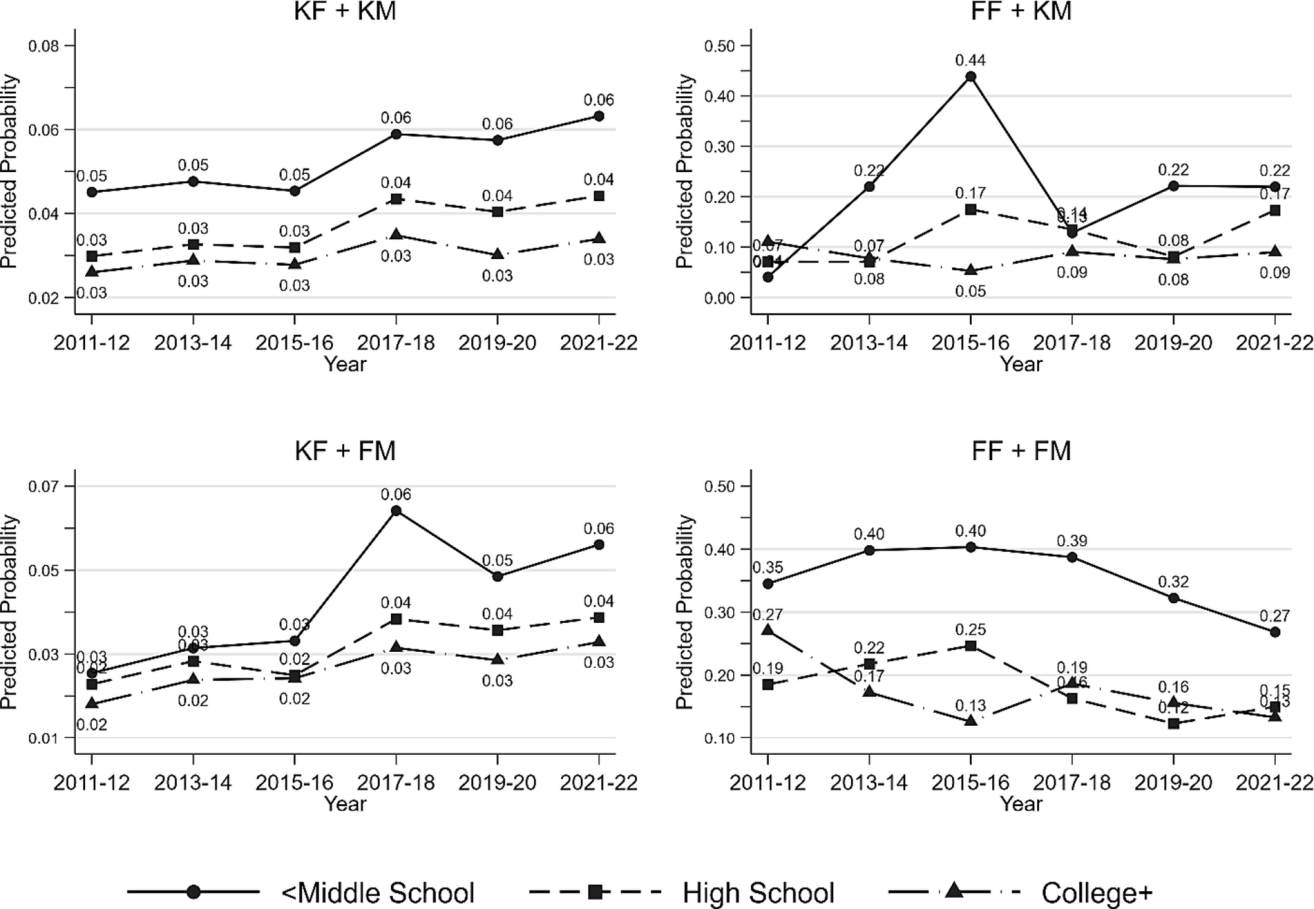
Predicted probabilities of sexual experience by family types, father’s Educational attainment, and periods. <Middle school, High school, and College+ denote “Middle School or Less,” “High School Graduates,” and “More than Junior College,” respectively. KF, KM, FF, and FM refer to “Korean Father,” “Korean Mother,” “Foreign Father,” and “Foreign Mother,” respectively. Predicted probabilities are calculated from coefficients in Model 2 in Table 2 by setting at mean values for all variables except family types, father’s educational attainment, and year.

Figure 2 illustrates the predicted probabilities of having sexual experience, by multicultural family type and periods, delineated by father’s educational attainment.^4^ This graph is derived from the estimated coefficients in Table 2, by calibrating all covariates (except the three just mentioned) to their mean values. Of note is that the predicted probabilities of sexual intercourse are much higher among multicultural youths with a foreign father (i.e., FF + KM and FF + FM) than other groups. The graph also suggests that, regardless of multicultural family backgrounds and periods, the level of the father’s educational attainment and the probability of adolescent’s sexual experience show a negative gradient. In other words: for all family types, as the father’s educational attainment increases, the children’s probability of having a sexual experience declined.

In addition, the gaps in the likelihood of sexual experience by father’s educational level appears to be widening among KF + KM and KF + FM groups. In the early 2010s, the gap in the predicted probability between fathers with less than a middle school education and those with more than a college education was roughly 0.01 among KF + KM and KF + FM groups. However, this gap widened to 0.03 in the early 2020s, resulting in a three-fold increase in one decade. By contrast, for FF + KM and FF + FM groups, the patterns in the disparities by father’s education level were curvilinear. That is, the gap in the probability of an adolescent having sexual experiences between the lowest education group and the highest group gradually grew from the early to the mid-2010s. After the mid-2010s, however, the discrepancies have narrowed. In particular, among adolescents with a foreign father and foreign mother, the differences between the fathers with the lowest level of education and those having a college education declined most rapidly. These results indicate that, while the probability of having sexual experience for youths with foreign fathers is still relatively higher than their peers with Korean fathers, the differential probabilities are narrowing.

## 5 Discussion and conclusion

Despite the rapid increase in the number of adolescents having multicultural backgrounds in Korea over recent decades, research on the antecedents of adolescents’ sexual initiation has been minimal. Moreover, because most studies have relied on cross-sectional analyses, how adolescents’ sexual behavior changed over time is largely overlooked. To overcome these limitations, using a nationally representative sample of adolescents in middle schools and high schools, this study examined the determinants of having sexual intercourse across four groups of adolescents classified by multicultural backgrounds. In particular, we analyzed trends in the likelihood of sexual contact since the early 2010s.

Results from logistic regressions predicting the likelihood of ever having sexual intercourse suggest that there are significant discrepancies across youths with different multicultural backgrounds. More specifically, even after accounting for pertinent risk factors, the odds of having sexual contact increased by 2.8 times for youths with foreign-born fathers and Korean mothers, compared with those in families with two Korean parents. When both father and mother are foreign-born, the odds of being sexually experienced increased by 4.7 times relative to those with two Korean parents. On the other hand, while the odds of having sexual experience among adolescents with a Korean father and foreign-born mother were significantly higher than their peers with both Korean parents, the same odds lost their statistical significance after controlling for the family’s socioeconomic factors such as parent’s educational attainment, whether the family is intact, and a subjective evaluation of academic performance. These results clearly suggest that a youth’s likelihood of having sexual experiences markedly rises when the youth’s father is foreign-born rather than a native Korean. Also, whereas adolescents with Korean fathers and foreign-born mothers also have a significantly higher probability of being sexually active, most of these influences are mediated by family’s socioeconomic status.

With a long tradition of a patriarchal culture, fathers rather than mothers are still considered the main breadwinner for the family in Korea (Brinton and Oh, 2019). Given that roughly one third of foreign-born fathers are originally from China or Viet Nam (Statistics Korea, 2023), results from the present study may imply that foreign-born fathers, regardless of mother’s nationality, are relatively inefficient in controlling their teenage children’s sexual behaviors largely due to lack of sociocultural resources. On the other hand, over the past decade, approximately one out of ten newly-married couples each year is a marriage between Korean men and foreign-born women (Kim, 2021), and because of a considerable increase in the prevalence of this form of multicultural family, it appears that discrepancies in the parental control for youth’s sexual intercourse compared to Korean native families are receding gradually over time.

Furthermore, with respect to the trend in adolescent’s sexual intercourse, results were largely consistent with Kim (2022). Coefficients from logistic regression models that included dummy variables of survey years indicated that the likelihood of adolescent’s sexual intercourse did not change much during the early 2010s. However, the likelihood began to rise in 2017, and it abruptly dropped in 2020 perhaps owing to various forms of quarantine rules for the COVID19. However, as Kim (2022) reported, it increased substantially since 2021 as the strict implementation of social distancing began to loosen.

Consistent with pertinent prior studies was the performance of certain risk factors for adolescent’s sexual intercourse. For example, male adolescents are significantly more likely to experience sexual intercourse than females, even after accounting for other factors. Also, as a teen’s age increases, the probability of having sexual intercourse increases monotonically. For example, other things being equal, 12th graders were about 5.8 times more likely to be sexually experienced than 7th graders.

On the other hand, some results did not support previous research. Hong and Kang (2017), for instance, claimed that the prevalence of sexual experiences is much higher among teens in coeducational schools than those in single-sex schools. However, the current results indicated that this likelihood is significantly higher in boys-only schools. This finding seems to be related to how the sexual experience is measured.

As with any research, the present study is not without limitations. First, some past literature suggested that peer influences have a substantial impact on the sexual initiation among adolescents (National Research Council, 1987); perceptions and norms in peer groups are closely associated with sexual behaviors among youths, and information about adolescent’s friends may be able to raise predictability on their sexual initiation. However, because of the data limitations in the KYRBS, the current study was unable to examine the effects of peer groups on adolescent’s sexual intercourse. An important avenue for future research should include sexual behaviors, as well as perceptions to them among an adolescent’s peers.

Second, prior studies on the association between immigrant status and sexual behaviors found that the duration of residence in the host country may have considerable influence on the likelihood of sexual contact (Blake et al., 2001). That is, a short duration of residence can be translated into lower levels of assimilation to the host country’s culture, and adolescents who are not sufficiently assimilated may start sexual relationship relatively later. Nevertheless, the KYRBS implicitly assumed that all students are born in Korea and did not ask about the year of entry to Korea for multicultural respondents. Due to the omission of the timing information, this study was unable to take the duration of residence into account.

Third, it is likely that different cultural backgrounds among multicultural adolescents might be related to the different levels of sexual experiences. That is, it is possible that adolescents whose parents are from countries more open to youth sexuality have earlier sexual experiences mainly through socialization. Thus, it would be meaningful to compare the odds of sexual experience by parents’ countries of origin. Owing to small number of adolescents from multicultural families but numerous parents’ countries of origin, however, it would be difficult to have reliable estimates by including a variable that represents parents’ countries of origin in a model. Nonetheless, future research should account for parents’ countries of origin to refine the effect of cultural heritage on multicultural adolescents’ sexual behaviors.

Finally, though the present study investigated the trends in sexual intercourse over time, it is not a longitudinal study in a strict sense. That is, the KYRBS is a repeated cross-sectional data, which sampled different groups of adolescents in every wave. Hence, we were not able to adequately account for the unobserved heterogeneity that can vary over time within an individual. Using panel data, future research should be able to address these limitations.

In sum, results from the present study suggest that, ultimately, parents can play a critical role in controlling their adolescent’s sexual behaviors and, in the case of multicultural families, it might be difficult to effectively provide sex education for their children due to cultural differences or a lack of socioeconomic resources. In this study, the father’s educational attainment had a strong effect on the sexual experiences of adolescents from multicultural families; the importance of a father’s participation in education should therefore be emphasized along with the mother’s role in the case of multicultural youths.

In addition, in the case of multicultural adolescents in Korea, there is not only a lack of basic sex education that must be done at home, but also a general lack of sex-related information outside of school, such as sex-related information provided between close friends. Even if sex education at school is not quite effective for native teenagers, it can be fruitful for teenagers from multicultural families. Therefore, sex education in schools should be streamlined to the needs and knowledge of multicultural adolescents.

Results from the current analysis imply that one of the fundamental drivers for earlier initiation of sexual activities among multicultural adolescents compared to their native peers can be attributable to motivational deficiencies due to their family’s limited socioeconomic resources. Hence, social policies for multicultural adolescents should be able to provide knowledge and means to enhance their self-esteem as well as to broaden life opportunities in Korean society.

## Data availability statement

Publicly available datasets were analyzed in this study. This data can be found at: https://www.kdca.go.kr/yhs/.

## Ethics statement

Ethical approval was not required for the study involving human samples in accordance with the local legislation and institutional requirements. Written informed consent for participation in this study was provided by the participants’ legal guardians/next of kin.

## Author contributions

KK: Writing – review & editing, Writing – original draft, Visualization, Validation, Supervision, Software, Methodology, Funding acquisition, Formal analysis, Data curation, Conceptualization. SJ: Writing – review & editing, Resources, Project administration.

## Footnotes

^1^In the 2011 and 2012 waves of the KYRBS, respondents were asked to categorize the experience as kiss, foreplay, coital penetration, or sexual assault. Based on this information, adolescents who engaged in sexual intercourse could be identified. However, beginning with the 2013 wave, these details about the sexual experience are not surveyed; respondents were instead asked whether they ever had sexual intercourse. Nevertheless, it is highly unlikely that the omission of specific forms of sexual experience since the 2013 wave would influence the analyses of this study because the share of respondents who reported other forms of sexual activity other than coital penetration were quite small (mostly less than 2%, except kiss).

^2^Ministry of Education (2022) defines the multicultural adolescents as adolescents from multicultural backgrounds, including those from international marriage households, households of foreign workers, and those who immigrated from foreign countries. Also, Ministry of Education (2022) determines as multicultural youth if one or both of adolescent’s parents does not hold Korean citizenship.

^3^Given the considerable refusal rate for questions about parent’s country of birth, preliminary analyses were performed using only 2011 to 2018 waves, which did not administer the agreement question. However, the results were almost identical as those presented in this paper. These results are available upon request.

^4^Results from logistic regression suggested that mother’s educational attainment is also closely associated with adolescent’s odds of having sexual experience. Furthermore, in general, mothers might have closer control of adolescents in Korean society than fathers. Nevertheless, we focused here on the effect of father’s education because family’s overall social class tends to be judged by father’s socioeconomic status rather than mother’s in Korea.
